# IgG4-related kidney disease (IgG4-RKD) with membranous nephropathy as its initial manifestation: report of one case and literature review

**DOI:** 10.1186/s12882-019-1419-6

**Published:** 2019-07-16

**Authors:** Nan-Nan Zhang, Yan-Yun Wang, Ling-Xin Kong, Wan-Zhong Zou, Bao Dong

**Affiliations:** 1 Department of Nephrology, Fangshan Hospital of Beijing University of Chinese Medicine, Beijing, 102400 China; 2 Department of Nephrology, People’s Hospital of Peking University, Beijing, 110102 China

**Keywords:** IgG4-related kidney disease, Interstitial nephritis, Membranous nephropathy, Repeated renal biopsies

## Abstract

**Background:**

IgG4-related disease (IgG4-RD) often affects multiple organs and tissues, especially the kidneys, and is characterized by interstitial nephritis, obstructive nephropathy, and in rare cases glomerulopathy (including membranous nephropathy).

**Case presentation:**

In this article, we report a patient with nephrotic syndrome as the only initial manifestation. Membranous nephropathy was confirmed by renal biopsy, but without any renal interstitial lesions. The nephrotic syndrome completely resolved after treatment with immunosuppressants but recurred after drug withdrawal, which was accompanied by acute kidney injury. Ultimately, IgG4-related interstitial nephritis with membranous nephropathy was confirmed by a second renal biopsy. After routine administration of steroids and cyclophosphamide, renal function returned to normal after 2 months, and nephrotic syndrome was ameliorated after 5 months.

**Conclusion:**

Special attention should be paid to this rare condition in the clinical setting. In patients with membranous nephropathy (MN) that is accompanied by multi-system damage, impaired renal function, elevated IgG4 levels (absolute or relative value), negative PLA2R, and/or renal interstitial plasma cell infiltration, the possibility of IgG4-related kidney disease (IgG4-RKD) should be carefully assessed.

## Background

Elevated serum IgG4 levels were found in patients with autoimmune pancreatitis (AIP) [1] in 2001, and in 2003, the concept of IgG4-related disease (IgG4-RD) was first proposed by Japanese authors [2]. Since then, IgG4-RD has gradually been recognized worldwide. IgG4-RD can affect one or multiple organs/tissues including meninges, salivary glands, lacrimal glands, lymph nodes, thyroid, lungs, mediastinum, biliary tract, pancreas, aorta, kidneys, bladder, skin, and nerves [3]. The affected organs/tissues have a similar manifestation, and infiltration of IgG4-positive plasma cells and hyper-IgG4-emia are found in most cases [4]. IgG4-RKD refers to the involvement of the kidney and its surrounding organs/tissues in IgG4-RD. The main feature of renal injury is IgG4-related tubulointerstitial nephritis (IgG4-TIN) [5, 6], accounting for 15–24.6% of all IgG-RD [5, 7]. IgG4-TIN can be accompanied by glomerular lesions or by chronic sclerosing inflammation of the lacrimal gland or salivary gland inflammation but without AIP; in rare cases, the lesions are limited only to the kidney [8]. Thus, the rates of misdiagnosis and missed diagnosis are often high. Here, we report the case of a patient with IgG4-RKD, with nephrotic syndrome as its initial manifestation. The first renal biopsy only showed MN, which was completely resolved after the combined use of steroids and cyclophosphamide. The nephrotic syndrome later recurred, accompanied by acute kidney injury (AKI). A second renal biopsy showed that both the renal interstitium and glomeruli were involved. Further histopathology confirmed the diagnosis of IgG4-TIN combined with MN; therefore, the treatment protocol was changed, with satisfactory therapeutic effectiveness. The evolution of this disease type has not been described previously in the literature.

## Case presentation

**Fig. 1:**
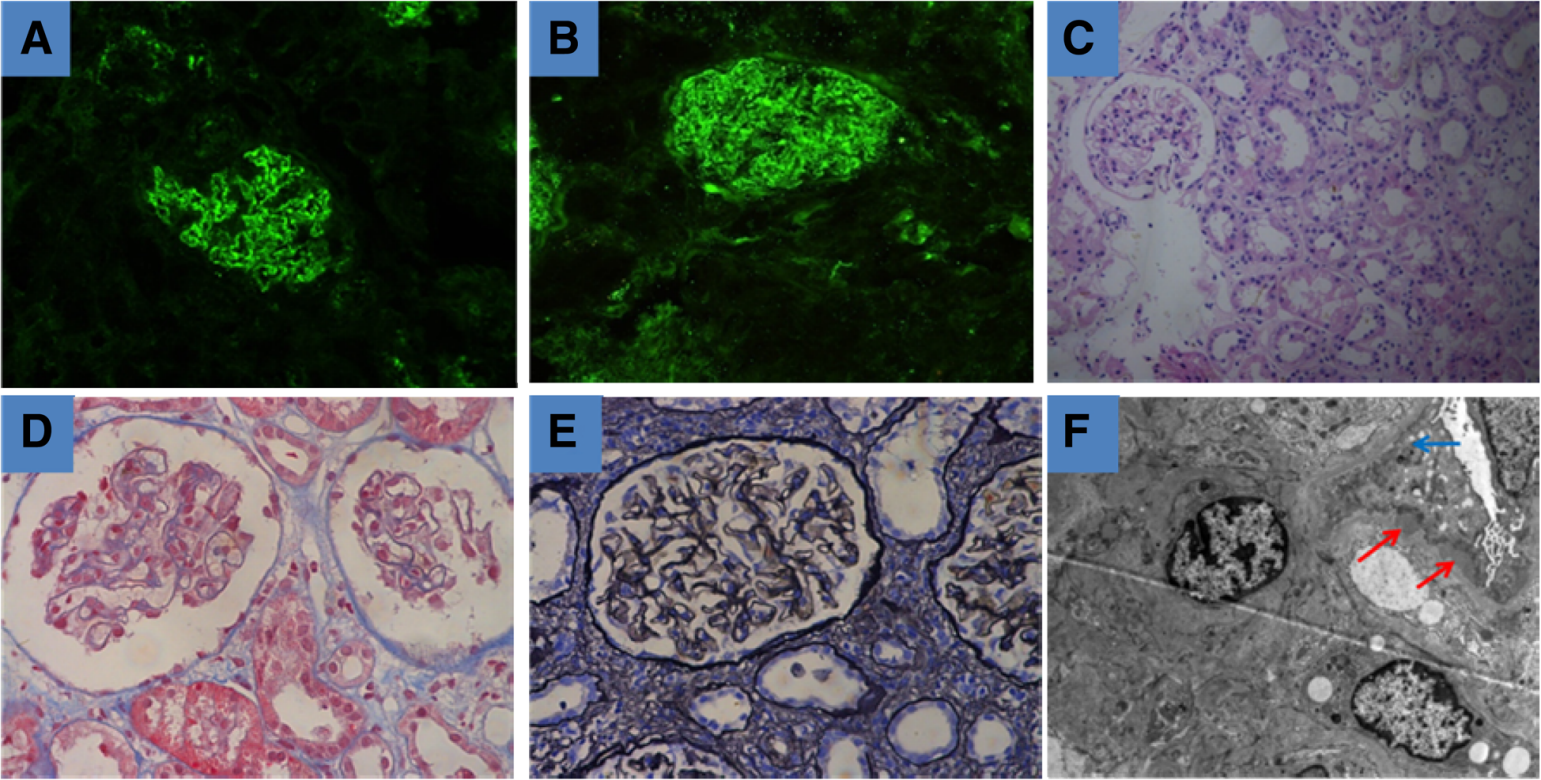
Histopathological findings on the first renal biopsy. **a** and **b** Particle-like deposition of IgG and C3, along the capillary wall (immunofluorescence assay). **c** No specific lesions in the renal interstitium (light microscopy: hematoxylin and eosin (HE), × 200). **d** and **e** Diffuse thickening of the glomerular basement membrane, subepithelial deposition of fuchsinophilic protein, and segmental spike formation (light microscopy: Masson and periodic acid-sliver methenamine(PASM), × 400). **f** Diffuse and irregular thickening of electron-dense deposits in the subepithelial, intrabasal, and mesangial areas and diffuse fusion of the foot processes among epithelial cells; however, no specific lesions were seen in renal tubules or interstitium. Red Arrow: electron-dense deposits; Blue arrow: diffuse fusion of the foot processes among epithelial cells (electron microscopy, × 6000)

A 46-year-old male patient was hospitalized in March 2015 because of bilateral lower extremity edema, and a diagnosis of nephrotic syndrome was made. The patient displayed evidence of chronic bilateral lacrimal gland inflammation, with exophthalmos, tearing, and bulbar conjunctival hyperemia. Laboratory test results are shown in Table 1. The immunofluorescence assay results from a renal biopsy were as follows: IgA−, IgG+++, IgM++, C1q−, and C3+++; and particle-like deposition was seen along the capillary wall. Light microscopy showed diffuse thickening of the glomerular basement membrane, subepithelial deposition of fuchsinophilic protein, segmental spike formation, vacuolar degeneration of renal tubules, and mild interstitial edema; however, while there was no infiltration of inflammatory cells, thickening of small arterial wall was observed. Electron microscopy showed the proliferation of glomerular mesangial cells and interstitial cells, diffuse and irregular thickening of basement membrane, electron-dense deposits in the subepithelial, intrabasal, and mesangial areas, and diffuse fusion of the foot processes among epithelial cells; however, no specific lesions were seen in renal tubules or the interstitium. All these findings met the diagnostic criteria for stage II MN (Fig. 1). While he was hospitalized, his serum creatinine increased progressively, his albumin levels were extremely low, the edema gradually worsened, and his urine output was low. To prevent further deterioration of renal function and secondary thrombosis, we administered prednisone acetate (60 mg/day for 8 weeks, which was then reduced by 5 mg every 4 weeks as the patient’s condition permitted) and cyclophosphamide (0.6 g/month by intravenous injection, to a total of 6 g). After 11 months of treatment, the patient’s condition was completely relieved. The prednisone acetate and cyclophosphamide were discontinued, and irbesartan was administered as maintenance therapy.

**Table 1:** Results of assays performed before and after treatment in the patient with IgG4-RKD

Time	First attack (March 2015)	After the first treatment (February 2016)	Second attack (August 2016)	Recent (March 2018)
Item				
Urinary protein/red blood cells	3+/+	−/−	3+/−	−/−
24-h urine protein (0.024–0.15 g/24 h)	5.226↑	0.665↑	11.78↑	1.308↑
Hemoglobin (110-170 g/L)	157	166	137	154
Eosinophils (0–0.3 × 10^9^/L)	0.07	0.05	0.02	0.09
Serum creatinine (40-120 μmol/L)	96	77	340↑	89
Total blood protein (60–80 g/L)	50.8↓	70	38.3↓	64
Albumin (35–55 g/L)	18.9↓	41.8	13.7↓	43.7
Serum IgG (8–16 g/L)	5↓	8.2	1.62↓	8.4
Serum IgG4 (3-201 mg/dl)			25.5	118
Serum C3 (0.8–1.6 g/L)	0.89	1.1	0.38↓	1.21
Serum C4 (0.2–0.4 g/L)	0.06↓	0.3	0.09↓	0.23
PLA2R	Negative		Negative	
Kidney sizes	Left kidney 12.7 cm × 5.0 cm, and right kidney 10.2 cm × 4.0 cm		Left kidney 13.3 cm × 5.8 cm, and right kidney 11.7 cm × 5.4 cm

**Fig. 2:**
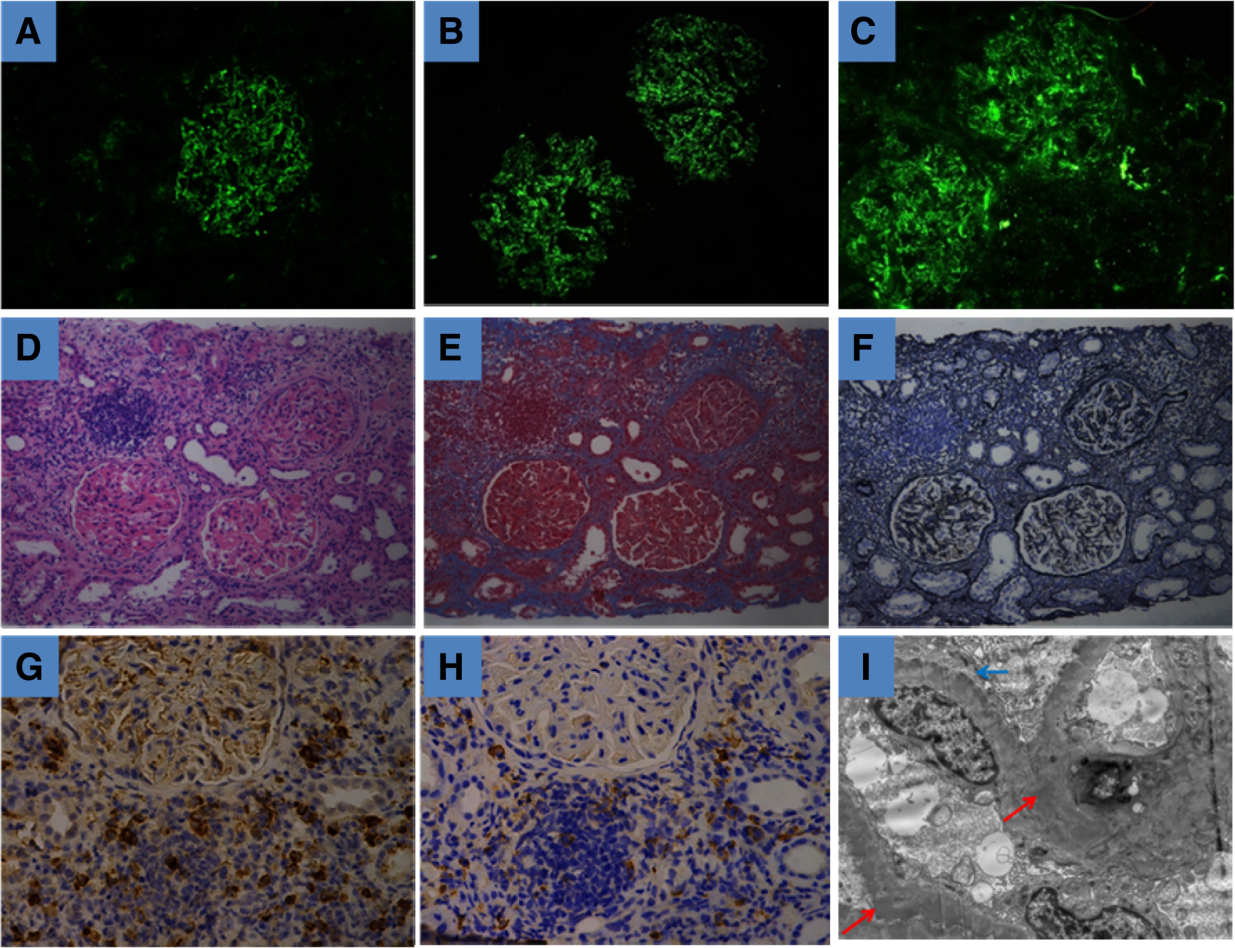
Pathological findings on the second renal biopsy. **a**, **b** and **c** Mass- and particle-like depositions of IgG, IgG4, and C3 along the mesangial area and capillary wall (immunofluorescence assay). **d**, **e** and **f** Mildly diffuse proliferation of glomerular mesangial cells and interstitial cells, moderate aggravation of focal segmental lesions, diffuse thickening of basement membrane (along with diffuse spike formation), subepithelial and mesangial deposition of fuchsinophilic protein, vacuolar and granular degeneration of renal tubular epithelial cells, multifocal loss of brush border, dilation of the renal tubules, multifocal atrophy, diffuse infiltration of lymphocytes, monocytes, and plasma cells in renal interstitium, multifocal fibrosis, and thickening of small arterial wall (light microscopy: HE, Masson, and PASM staining, × 200). **g** and **h** Immunohistochemical staining of IgG and IgG4. **i** Proliferation of glomerular mesangial cells and interstitial cells, diffuse and irregular thickening of basement membrane, electron-dense deposits in the subepithelial, intrabasal, subendothelial, and mesangial areas, diffuse fusion of the foot processes among epithelial cells, detachment and partial atrophy of the microvilli of renal tubular epithelial cells as well as edema, infiltration of lymphocytes/monocytes, and fibrosis in renal interstitium. Red Arrow: electron-dense deposits; Blue arrow: diffuse fusion of the foot processes among epithelial cells(electron microscopy, × 6000)

In August 2016, the patient was re-hospitalized because of nephrotic syndrome recurrence, accompanied by AKI. The results of relevant tests are shown in Table 1. Considering that AKI is seldom caused by MN, we performed another renal biopsy. Immunofluorescence assay results were as follows: IgA−, IgG+++, IgM++, C1q−, and C3+++, and mass- and particle-like depositions were seen along the mesangial area and capillary wall. Light microscopy showed the mildly diffuse proliferation of glomerular mesangial cells and interstitial cells, moderate aggravation of focal segmental lesions, diffuse thickening of basement membrane (together with diffuse spike formation), subepithelial deposition of fuchsinophilic protein, vacuolar and granular degeneration of renal tubular epithelial cells, multifocal loss of brush border, dilation of the renal tubules, multifocal atrophy, diffuse infiltration of lymphocytes, monocytes, plasma cells in the renal interstitium, multifocal fibrosis, and thickening of small arterial walls. Electron microscopy showed the proliferation of glomerular mesangial cells and interstitial cells, diffuse and irregular thickening of basement membrane, electron-dense deposits in the subepithelial, intrabasal, subendothelial, and mesangial areas, diffuse fusion of the foot processes among epithelial cells, detachment and partial atrophy of the microvilli of renal tubular epithelial cells as well as edema, infiltration of lymphocytes/monocytes, and fibrosis in the renal interstitium. Immunohistochemistry revealed CD38^+^, CD138^+^, IgG^+^, and IgG4^+^ cells (approximately 30% of IgG^+^ cells) (Fig. 2). Based on the immunohistochemical findings, clinical manifestations, and exclusion of other secondary factors, a diagnosis of IgG4-TIN accompanied by MN was made.

**Fig. 3:**
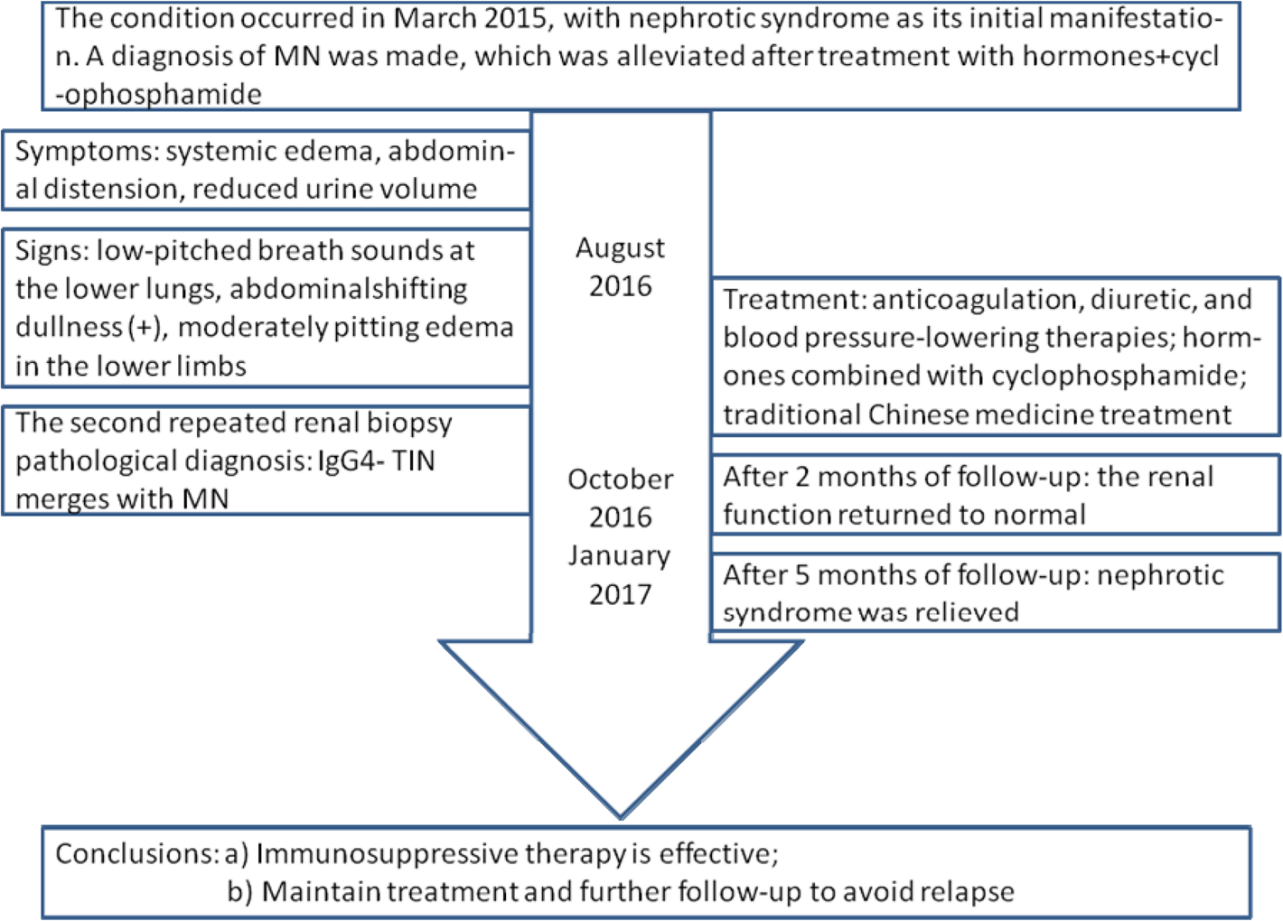
Flow diagram of the patient’s disease progression and treatment

The patient had acute kidney injury and was in critical condition. Prednisone acetate and cyclophosphamide were administered in accordance with the previous treatment regimen. A further 6 g of cyclophosphamide was then administered (to a total of 12 g), and prednisone acetate was continued at 10 mg/day for maintenance. His renal function returned to normal after 2 months, and nephrotic syndrome was ameliorated after 5 months. The patient has been closely followed-up to date (Fig. 3).

## Discussion and conclusions

IgG4-RKD is any form of renal involvement in IgG4-RD [4]. Its typical manifestation is TIN with multiple organ damage [5, 7], which can be accompanied by a chronic or rapid progressive decline in renal function [9, 10]. Only a small number of IgG4-RKD patients have disease that involved only the glomeruli and that was initially manifested as nephrotic syndrome, and approximately 7% of IgG4-RD patients have undergone renal biopsy [11]. The number of patients with disease recurrence accompanied by IgG4-TIN is even smaller.

Our current patient had an initial disease manifestation of MN, which manifested as nephrotic syndrome. The disease was alleviated after treatment but relapsed upon drug withdrawal, and renal insufficiency was very serious. After the second renal biopsy, a pathological diagnosis of IgG4-TIN with MN was made. Comparison of the results of the two renal biopsies confirmed that the MN found during the first attack was also associated with IgG4-RKD. Thus, IgG4-RKD can be initially manifested as MN, followed by TIN. IgG4-RD is a cause of secondary MN [4]. This extremely rare form of IgG4-RKD features initial involvement of renal glomeruli, followed by the renal interstitium as the disease progresses. While IgG4-RKD with TIN as its initial manifestation will not be missed, IgG4-RKD with MN as its initial manifestation can be easily overlooked. Therefore, this special form of IgG4-MN should be considered to be a differential diagnosis in patients presenting with MN, to ensure the early identification of IgG4-RD and avoid a missed diagnosis and delayed treatment.

How can this early identification of IgG4-MN in patients with MN be achieved? First, MN patients often have multi-system injuries, in which the possibility of IgG4-RKD involvement should be identified. In our current case, during the first disease attack, the patient had unexplained binocular chronic lacrimal gland inflammation. He also had exophthalmos, epiphora, and congestive conjunctiva. Chronic dacryocystitis is an infection of the lacrimal sac that is typically associated with nasolacrimal duct obstruction [12], which was an important clinical clue. Second, laboratory tests showed that renal dysfunction progressed slowly in the patient. If patients with MN have AKI after recurrence or during treatment, a change in the pathological type should be considered after factors such as pre-renal azotemia, thrombosis, and medications are ruled out. A second renal biopsy should be performed to avoid a missed diagnosis of IgG4-MN. The typical laboratory findings of IgG4-MN include hyper-IgG-emia, hyper-IgG4-emia, and hyper-IgE-emia [4]. In our current case, although the total IgG level was not high, the relative level of IgG4 was markedly elevated, which might be caused by proteinuria and massive loss of IgG in the patient. Although the relative serum levels of IgG and IgG4 were not high, the proportion of IgG4 was high over the total IgG, which was also highly suggestive of IgG4-MN. The serum anti-M-type phospholipase A2 receptor (PLA2R) is typically positive in primary MN patients, but it is negative in patients with MN secondary to IgG4-RD [13–15]. A meta-analysis showed that the sensitivity and specificity of serum anti-PLA2R antibody were 68 and 97% in the differential diagnosis of primary MN and secondary MN, respectively [16]. In our current case, PLA2R was negative during both attacks. Thus, patients with PLA2R-negative MN should be further examined to identify IgG4-RKD and other secondary MN. In addition, primary MN has similar pathological findings to IgG4-MN. The typical renal pathological features of IgG4-RKD include renal interstitial fibrosis and focal lymphoplasmacytic infiltration, during which eosinophil infiltration, tubulitis, and inflammatory cell infiltration of the renal capsule can be observed. Immunohistochemistry shows interstitial IgG4+ plasma cell infiltration (> 10/hpf), and IgG4-positive plasma cells account for more than 40% of IgG+ plasma cells [13, 15]. Clinicians treating MN patients presenting with inflammatory cell infiltration and tubule-interstitial injury also require special staining to facilitate the early detection of IgG4-RKD. While IgG-RKD can also be treated using the standard combination of immunosuppressant and steroid therapy [9, 10], it has a higher recurrence rate than MN [17], and delayed treatment will increase the risk of renal failure [18]. The differences between primary MN and IgG4-MN are summarized in Table 2.

**Table 2:** Differences between primary MN and IgG4-MN

Item		Primary MN	IgG4-MN
Clinical manifestations		Often without damaging other systems	Other with multi-system injuries including lacrimal gland inflammation, salivary gland inflammation, and pancreatitis
Laboratory tests	Renal function	Often normal	Often abnormal
	Serum IgG4	Often not elevated	(Absolute or relative values) often elevated
	Serum IgE	Often not elevated	Often elevated
	PLA2R	Often positive	Negative
Pathology	Pathological IgG subtypes	Various	Mainly IgG4
	Interstitial damage	Without plasma cell infiltration and often without interstitial damage	With plasma cell infiltration and often with interstitial damage
Treatment protocol	Hormone dosage	Typically adequate (Prednisone dose that was 1–2 mg/kg/d)	Generally medium and small dose (Prednisone dose that induced was 30–40 mg/d)
	Withdrawal of hormone	Hormone are withdrawn regularly, and will be stopped when the condition is alleviated	Maintenance is required

TIN is the most common initial manifestation of IgG4-RKD, whereas IgG4-RKD with MN as its initial manifestation is much rarer. In patients with MN accompanied by multi-system damage, impaired renal function, elevated IgG4 (absolute or relative value), negative PLA2R, and/or renal interstitial plasma cell infiltration, the possibility of IgG4-RKD should be carefully assessed.

## Data Availability

All data and materials are provided by formal institutions, and they are valid.

## Abbreviations

AIP: Autoimmune pancreatitis
AKI: Acute kidney injury
IgG4-RD: IgG4-related disease
IgG4-RKD: IgG4-related kidney disease
IgG4-TIN: IgG4-related tubulointerstitial nephritis
MN: Membranous nephropathy
PLA2R: Phospholipase A2 receptor

## References

[1] Hamano H, Kawa S, Horiuchi A (2001). High serum IgG4 concentrations in patients with sclerosing pancreatitis. N Engl J Med.

[2] Kamisawa T, Okamoto A (2008). IgG4-related sclerosing disease. World J Gastroenterol.

[3] Kawano M, Saeki T (2015). IgG4-related kidney disease-an update. Curr Opin Nephrol Hypertens.

[4] Zheng K, Teng F, Li X-M (2017). Immunoglobulin G4-related kidney disease:Pathogenesis,diagnosis,and treatment. Chronic Dis Transl Med.

[5] Saeki T, Nishi S, Imai N (2010). Clinicopathological characteristics of patients with IgG4-related tubulointerstitial nephritis[J]. Kidney Int.

[6] Yamaguchi Y, Kanetsuna Y, Honda K (2012). Characteristics tubulointerstitial nephritis in IgG4-related disease[J]. Hum Pathol.

[7] Lin W, Lu S, Chen H (2015). Clinical characteristics of immunoglobulin G4-related disease:a prospective study of 118 Chinese patients. Rheumatol(Oxford).

[8] Shoji S, Nakano M, Usui Y (2010). IgG4-related infl ammatory pseudotumor of the kidney[J]. Int J Urol.

[9] Zheng K, Li XM, Cai JF, Wen YB (2012). Analysis on urinary system lesions of IgG4-related disease. Chin J Nephrol.

[10] Chen G, Zheng K, Ye WL (2015). Clinical feature of renal impairment caused by IgG4 related disease with renal and urinary lesions. Chin J Nephrol.

[11] Cornell LD (2012). IgG4-related kidney disease. Curr Opin Nephrol Hypertens.

[12] Machado MAC, Silva JAF, Garcia EA, Allemann N (2017). Ultrasound parameters of normal lacrimal sac and chronic dacryocystitis. Arq Bras Oftalmol.

[13] Alexander MP, Larsen CP, Gibson IW (2013). Membranous glomerulonephritis is a manifestation of IgG4-related disease. Kidney Int.

[14] Khosroshahi A, Ayalon R, Beck LH, Salant DJ, Bloch DB, Stone JH (2012). IgG4-related disease is not associated with antibody to the phospholipase A2 receptor. Int J Rheumatol.

[15] Wada Y, Saeki T, Yoshita K (2013). Development of IgG4-related disease in a patient diagnosed with idiopathic membranous nephropathy. Clin Kidney J.

[16] Dai H, Zhang H, He Y (2015). Diagnosic accuracy of PLA2R autoantibodies and glomerular staining for the differentiation ofidiopathic and secondary membranous nephropathy:an updated meta-analysis[J]. Sci Rep.

[17] Khosroshahi A, Stone JH (2011). Treatment approaches to IgG4-related syetemic disease[J]. Curr Opin Rheumatol.

[18] Saida Y, Homma M-N, Hama H (2010). A case of IgG4-related tubulointerstitial nephritis showing the progression of renal dysfunction after a cure for autoimmue pancreatitis. Nihon Jinzo Gakkai Shi.

